# Methionine restriction restores a younger metabolic phenotype in adult mice with alterations in fibroblast growth factor 21

**DOI:** 10.1111/acel.12238

**Published:** 2014-06-17

**Authors:** Emma K Lees, Elżbieta Król, Louise Grant, Kirsty Shearer, Cathy Wyse, Eleanor Moncur, Aleksandra S Bykowska, Nimesh Mody, Thomas W Gettys, Mirela Delibegovic

**Affiliations:** 1 Institute of Medical Sciences, College of Life Sciences and Medicine, University of AberdeenAberdeen, AB25 2ZD, UK; 2 Institute of Biological and Environmental Sciences, University of AberdeenAberdeen, AB24 2TZ, UK; 3 Nutrient Sensing and Adipocyte Signaling Department, Pennington Biomedical Research CenterBaton Rouge, LA, 70808, USA; * Institute of Biodiversity, Animal Health and Comparative Medicine, University of GlasgowGraham Kerr Building, Glasgow, G12 8QQ, UK

**Keywords:** activating transcription factor 4, aging, fibroblast growth factor 21, lipid, metabolism, unfolded protein response

## Abstract

Methionine restriction (MR) decreases body weight and adiposity and improves glucose homeostasis in rodents. Similar to caloric restriction, MR extends lifespan, but is accompanied by increased food intake and energy expenditure. Most studies have examined MR in young animals; therefore, the aim of this study was to investigate the ability of MR to reverse age-induced obesity and insulin resistance in adult animals. Male C57BL/6J mice aged 2 and 12 months old were fed MR (0.172% methionine) or control diet (0.86% methionine) for 8 weeks or 48 h. Food intake and whole-body physiology were assessed and serum/tissues analyzed biochemically. Methionine restriction in 12-month-old mice completely reversed age-induced alterations in body weight, adiposity, physical activity, and glucose tolerance to the levels measured in healthy 2-month-old control-fed mice. This was despite a significant increase in food intake in 12-month-old MR-fed mice. Methionine restriction decreased hepatic lipogenic gene expression and caused a remodeling of lipid metabolism in white adipose tissue, alongside increased insulin-induced phosphorylation of the insulin receptor (IR) and Akt in peripheral tissues. Mice restricted of methionine exhibited increased circulating and hepatic gene expression levels of FGF21, phosphorylation of eIF2a, and expression of ATF4, with a concomitant decrease in IRE1α phosphorylation. Short-term 48-h MR treatment increased hepatic FGF21 expression/secretion and insulin signaling and improved whole-body glucose homeostasis without affecting body weight. Our findings suggest that MR feeding can reverse the negative effects of aging on body mass, adiposity, and insulin resistance through an FGF21 mechanism. These findings implicate MR dietary intervention as a viable therapy for age-induced metabolic syndrome in adult humans.

## Introduction

Aging is characterized by increased adiposity (Huffman & Barzilai, 2009) and insulin resistance (Selman & Withers, 2011), which may play a role in regulating lifespan (Huffman & Barzilai, 2009; Selman & Withers, 2011) due to their association with further metabolic complications, including type 2 diabetes, cardiovascular disease and cancer (Biddinger, 2006). Removal of visceral fat is enough to increase mean and maximum lifespan in rodents (Muzumdar *et al*., 2008). Visceral fat removal also improves insulin sensitivity in rats (Barzilai *et al*., 1999) and enhanced insulin sensitivity is a characteristic of many long-lived mouse models (Selman & Withers, 2011).

Methionine restriction (MR) is a dietary technique, with the only manipulation of the diet being a reduction in the essential amino acid methionine (from 0.86% of the diet to 0.172%). Methionine restriction has been shown previously to extend lifespan (Orentreich *et al*., 1993; Richie *et al*., 1994), dramatically decrease body weight and adiposity, and improve insulin sensitivity relative to animals on a control diet (Hasek *et al*., 2010; Plaisance *et al*., 2010; Ables *et al*., 2012). Methionine restriction has, therefore, been proposed to mimic effects of caloric restriction (CR) (Masoro, 2005); however, in contrast to CR, animals on MR diet are fed *ad libitum* and actually consume more food than control-fed animals (Hasek *et al*., 2010; Plaisance *et al*., 2010). This loss in body mass despite an increase in energy intake is thought to be accomplished through creating a vast metabolic inefficiency, which leads to increased energy expenditure, through uncoupling protein 1 (UCP1) nonshivering thermogenesis in adipose tissue (Hasek *et al*., 2010). In young animals, MR stunts growth and development, including reducing total length, serum insulin-like growth factor 1 (IGF-1), and growth hormone signaling (Ables *et al*., 2012). Fibroblast growth factor (FGF) 21 is another regulator of growth that is released from the liver in response to fasting through a PPARα mechanism (Badman *et al*., 2007). FGF21 transgenic mice also show decreased circulating levels of IGF-1 and are smaller than wild-type mice (Inagaki *et al*., 2008).

Insulin responsiveness in tissues depends on insulin binding to the insulin receptor (IR) on the surface of cells (Taniguchi *et al*., 2006). The receptor autophosphorylates itself and, therefore, stimulates its own tyrosine kinase activity, enabling it to phosphorylate and activate downstream proteins, including the insulin receptor substrates (IRS) and protein kinase B (PKB/Akt) to promote glucose uptake (Taniguchi *et al*., 2006). Akt also stimulates the mechanistic target of rapamycin complex 1 (mTORC1), which activates 4E binding protein 1 (4E-BP1) and p70 ribosomal S6 kinase (p70S6K). p70S6K phosphorylates ribosomal protein S6 (S6) to promote protein synthesis (Taniguchi *et al*., 2006). The endoplasmic reticulum (ER) is responsible for protein folding and maturation and in response to an overload of mis/unfolded proteins, due to increased demands for protein synthesis, becomes overactivated/stressed (Hotamisligil, 2010). In response to this stress, the unfolded protein response (UPR) is induced (ER stress) and involves three signaling branches, beginning with ER membrane-associated proteins; activating transcription factor-6 (ATF6), inositol requiring enzyme 1 α (IRE1α), and PKR-like endoplasmic reticulum kinase (PERK) (Hotamisligil, 2010).

Initiation of dietary MR early in life limits postweaning growth and tissue deposition, but in most studies to date, regardless of duration, the MR diet was provided continuously from a young age (Hasek *et al*., 2010; Perrone *et al*., 2010). In contrast, the impact of initiating MR at different ages in mice has not been systematically evaluated. As obese adults are the main target for MR intervention in humans (Plaisance *et al*., 2011), we aimed to examine the effectiveness of beginning MR at different ages, including adult mice (2 and 12 months old), to investigate whether it can reverse metabolic syndrome, and further identify whether it would make a suitable therapy for adult insulin-resistant humans. In molecular analyses, we focused on altered hepatic and white adipose tissue (WAT) signaling pathways, as MR has been shown, through transcriptional analysis, to exert its main action in these two tissues (Hasek *et al*., 2013).

## Results

### MR decreases body weight and fat mass, but increases food intake, glucose tolerance, and levels of physical activity

**Figure 1:**
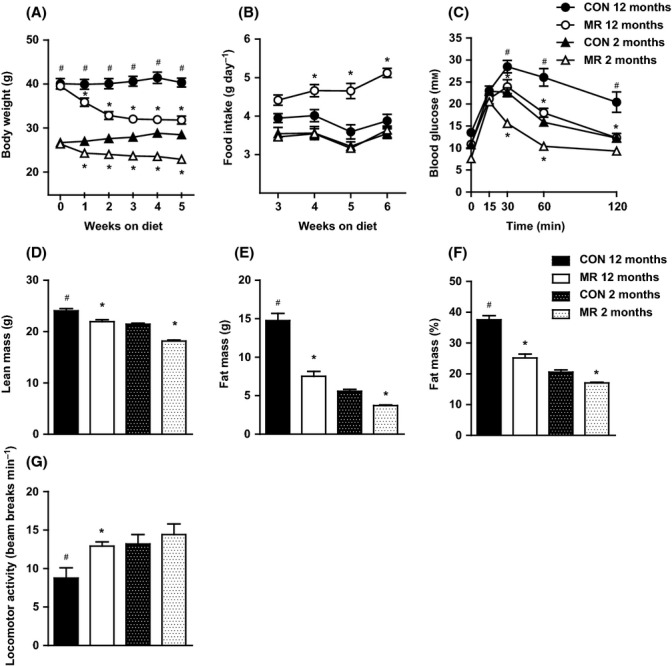
Body weight, food intake, glucose tolerance, lean mass, fat mass, and physical activity levels of mice on methionine restriction (MR) and control diet. (A) Body weight measurements from the first 5 weeks of the study, (B) food intake from a representative 4-week period of the study, and (C) glucose tolerance as assessed by a glucose tolerance test (dose of glucose = 2 g kg^−1^) after a 5-h fast in mice fed MR or control diet at the ages of 2 months (*n* = 20) and 12 months (*n* = 14). Significance was calculated by repeated measures two-way ANOVA with Bonferroni multiple comparison *post hoc* tests. (D) Lean mass (g), (E) fat mass (g), and (F) % fat mass of total body mass were measured by dual-energy X-ray absorptiometry in mice fed MR or control diet at the ages of 2 months (*n* = 20) and 12 months (*n* = 14). Significance was calculated by one-way ANOVA and Tukey’s *post hoc* test. (G) Physical activity was measured by beam breaks per minute in mice fed MR or control diet at the ages of 2 months (*n* = 6) and 12 months (*n* = 6). Significance was calculated by two-tailed Student’s *t*-test. Data are represented as mean ± SEM. Black bars/circles, 12-month-old control-fed mice; white bars/circles, 12-month-old MR-fed mice; black hatch bars/black triangles, 2-month-old control-fed mice; white hatch bars/white triangles, 2-month-old MR-fed mice. *MR-fed mice significantly different to age-matched control-fed mice (*P* < 0.05), #12-month-old control-fed mice significantly different to 2-month-old control-fed mice (*P* < 0.05).

To examine the effectiveness of MR when beginning the diet straight after weaning and when mice are fully grown and developed, the responses to 8 weeks of dietary MR were evaluated in C57BL/6J male mice in which the diet was initiated at 2 and 12 months of age. There was a significant increase in body weight from the 2-month- to the 12-month-old control-fed mice (Fig. 1A). Methionine restriction was able to significantly reverse this effect in the 12-month-old mice by decreasing body weight by 7.8 g by week 5 of dietary treatment (Fig. 1A). Interestingly, by week 5, the body weight of 12-month-old MR-fed mice reached a similar level to 2-month-old control-fed mice (31.6 vs. 28.3 g, respectively) (Fig. 1A). The control diet in the 12-month-old mice had little effect on body weight, which stayed stable at a 0.3-g increase throughout the study (Fig. 1A). In contrast, in young 2-month-old mice, the control diet produced a steady increase in body weight throughout the study due to growth (1.8 g), but MR prevented this and produced a significantly stable body weight loss, losing 3.5 g by week 5 of dietary treatment (Fig. 1A).

Food intake was not different between the diets for 2-month-old mice (Fig. 1B); however, the MR-fed mice weighed significantly less at every week throughout the study (Fig. 1A); therefore, the body weight adjusted food intake (g day^−1^ per BW) for 2-month-old MR-fed animals was significantly higher than 2-month-old control-fed mice (data not shown). Young MR-fed animals (2 months) were able to sustain a smaller body weight than control-fed animals by consuming the same amount of food (Fig. 1A,B). In 12-month-old mice, food intake (g day^−1^) was significantly increased by MR (Fig. 1B), so MR-fed mice were able to maintain a lower body weight with higher food consumption (Fig. 1A,B).

We examined the ability of MR to affect age-dependent changes in biomarkers of metabolic health in 2- and 12-month-old mice. Diminished glucose tolerance was present in 12-month- compared with 2-month-old control-fed mice (Fig. 1C). Methionine restriction significantly improved this effect on glucose excursion in 12-month-old mice and reached the same level of blood glucose response, at all time points, as healthy 2-month-old control-fed mice (Fig. 1C). Methionine restriction also significantly enhanced glucose tolerance in 2-month-old mice relative to their age-matched controls (Fig. 1C).

Lean mass (g) was significantly increased with age in control-fed mice and decreased by MR diet (Fig. 1D), but this is merely a reflection of the differences in total body weight (Fig. 1A), with % lean mass actually significantly decreased in 12-month-old compared with 2-month-old control-fed mice and significantly increased by MR relative to age-matched controls (data not shown). Fat mass (total g and %) significantly increased with age in control-fed mice (Fig. 1E,F). Methionine restriction was able to reverse this effect in 12-month-old mice by significantly decreasing fat mass to a comparable level of 2-month-old control-fed mice (Fig. 1E,F). Furthermore, MR significantly reduced the fat mass (g and %) of 2-month-old MR-fed mice relative to age-matched control-fed mice (Fig. 1E,F).

We also examined the effect of MR diet on physical activity levels. Physical activity levels significantly decreased with age in control-fed mice (Fig. 1G). Within 12-month-old mice, MR significantly increased activity levels, making the activity levels of 12-month-old MR-fed mice (12.9 beam breaks min^−1^) equivalent to that of 2-month-old control-fed mice (13.2 beam breaks min^−1^) (Fig. 1G).

### MR improves glucose and lipid metabolism

**Figure 2:**
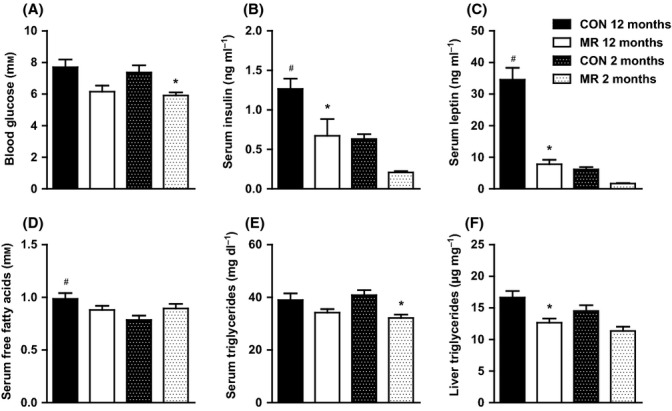
Whole-body glucose and lipid homeostasis of mice on methionine restriction (MR) and control diet. (A) Fasting blood glucose, (B) fasting serum insulin, (C) fasting serum leptin, (D) fasting serum FFAs, and (E) fasting serum triglycerides in mice fed MR or control diet at the ages of 2 months (*n* = 16–20) and 12 months (*n* = 14). (F) Liver triglycerides in mice fed MR or control diet at the ages of 2 months (*n* = 10) and 12 months (*n* = 10). Significance was calculated by one-way ANOVA and Tukey’s *post hoc* test. Data are represented as mean ± SEM. Black bars, 12-month-old control-fed mice; white bars, 12-month-old MR-fed mice; black hatch bars, 2-month-old control-fed mice; white hatch bars, 2-month-old MR-fed mice. *MR-fed mice significantly different to age-matched control-fed mice (*P* < 0.05), #12-month-old control-fed mice significantly different to 2-month-old control-fed mice (*P* < 0.05).

Due to the substantial improvements in glucose tolerance by MR diet, we went onto measure metabolic markers in the serum. Although MR decreased fasting blood glucose levels in both age groups, this was only significant in the 2-month-old mice (Fig. 2A). Aging had no effect on fasting blood glucose levels but produced a significant rise in fasting serum insulin levels (hyperinsulinemia) between 2- and 12-month-old control-fed mice (Fig. 2B). Methionine restriction was able to decrease fasting serum insulin levels significantly in 12-month-old mice, attaining the level of 2-month-old control-fed mice (Fig. 2B). In 2-month-old mice, fasting serum insulin levels were reduced by MR diet, but this was not significant (Fig. 2B). Aging significantly increased serum leptin levels between 2- and 12–month-old control-fed mice (Fig. 2C), but MR lowered serum leptin levels by approximately threefold in both age groups (Fig. 2C). Aging also increased fasting serum levels of free fatty acids (FFAs) in control-fed mice (12 vs. 2 months old); however, diet failed to alter FFAs at either age (Fig. 2D). Aging had no effect on serum or hepatic triglyceride levels (Fig. 2E,F); however, MR significantly decreased serum triglyceride levels in 2-month-old mice, but had no effect in 12-month-old mice (Fig. 2E) and decreased hepatic triglycerides in 12-month-old mice, but had no effect in 2-month-old mice (Fig. 2F).

### MR increases lipogenic and oxidative gene expression in epididymal WAT and decreases lipogenic gene expression in liver

**Figure 3:**
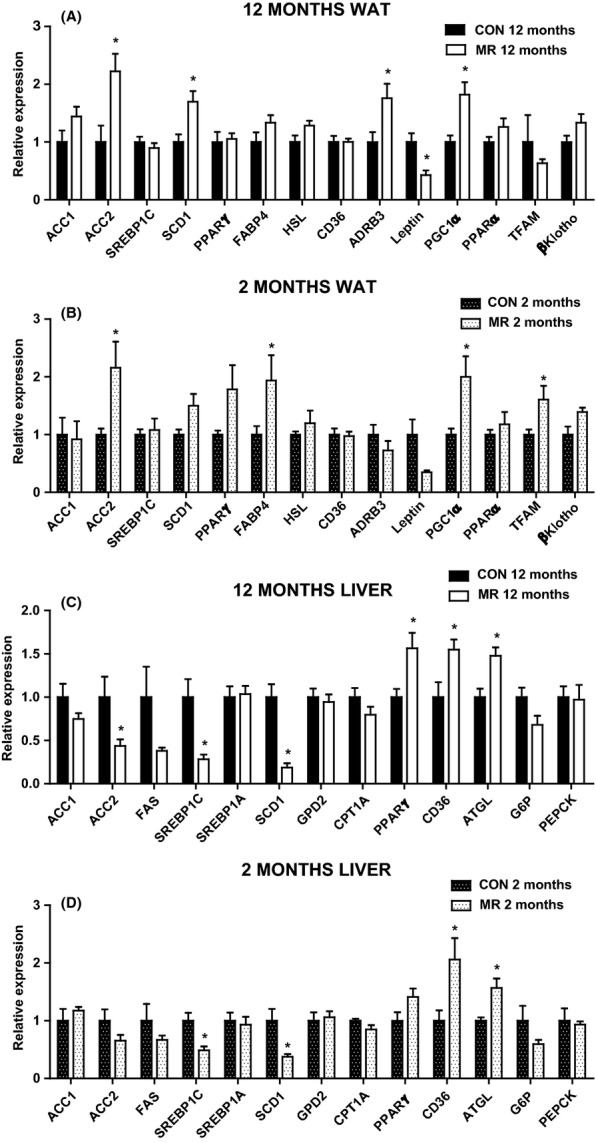
Epididymal white adipose tissue (WAT) and hepatic gene expression in mice on methionine restriction (MR) and control diet. Gene expression was measured by qPCR in (A) epididymal WAT in 12-month-old mice (*n* = 5–6), (B) epididymal WAT in 2-month-old mice (*n* = 4–6), (C) liver in 12-month-old mice (*n* = 5–8) and (D) liver in 2-month-old mice (*n* = 4–6) fed MR or control diet. Data were analyzed as fold change relative to control-fed mice. Significance was calculated between MR and control diet by two-tailed Student’s *t*-test (*P < 0.05). Data are represented as mean ± SEM. Black bars, 12-month-old control-fed mice; white bars, 12-month-old MR-fed mice; black hatch bars, 2-month-old control-fed mice; white hatch bars, 2-month-old MR-fed mice.

Diet-induced changes in circulating and tissue lipid levels suggest that dietary MR may be modifying tissue-specific synthesis, export, uptake, and/or oxidation of lipid. To explore these possibilities in greater detail, changes in expression of genes involved in different aspects of lipid metabolism were evaluated. In 12-month-old mice, MR had no effect on the expression of lipogenic and adipogenic promoting genes; *acetyl*-c*oA carboxylase 1* (*ACC1*) and *sterol regulatory element-binding protein 1c* (*SREBP1c*) in epididymal WAT (Fig. 3A). However, MR significantly upregulated *acetyl*-c*oA carboxylase 2* (*ACC2*) and *stearoyl-CoA desaturase-1* (*SCD1*) (Fig. 3A). Dietary treatment had no effect in 12-month-old mice on *peroxisome proliferator-activated receptor gamma* (*PPAR*γ), *fatty acid-binding protein 4* (*FABP4*), *hormone-sensitive lipase* (*HSL*), and *cluster of differentiation 36* (*CD36*) (Fig. 3A). Methionine restriction significantly upregulated *beta-3 adrenergic receptor* (*ADRB3*) in 12-month-old mice and in parallel with the decreased circulating leptin levels, significantly decreased expression of *leptin* (Fig. 3A). *Peroxisome proliferator-activated receptor gamma coactivator 1-alpha* (*PGC-1*α), a master regulator of mitochondrial biogenesis, was increased significantly by MR in WAT in 12-month-old mice, but *peroxisome proliferator-activated receptor alpha* (*PPAR*α) levels were unchanged (Fig. 3A). Levels of *mitochondrial transcription factor A* (*TFAM*) were unchanged by MR, but MR upregulated β-*Klotho* in 12-month-old mice; however, this was not significant (Fig. 3A).

Methionine restriction produced similar changes to gene expression levels in epididymal WAT in 2-month-old mice compared with the 12-month-old mice (Fig. 3B). However, *SCD1* was not changed by diet, but *FABP4* was significantly upregulated and *PPAR*γ was also increased (*P* = 0.05) by MR (Fig. 3B). *ADRB3* and *leptin* gene expression levels were also not different between diets in 2-month-old mice, but *TFAM* was significantly increased by MR (Fig. 3B).

In the liver, MR produced a significant downregulation of the lipogenic genes, *ACC2*, *SREBP1c,* and *SCD1* in 12-month-old mice (Fig. 3C). *Acetyl*-c*oA carboxylase 1*, *fatty acid synthase* (*FAS*), *SREBP1*α, *glycerol-3-phosphate dehydrogenase* (*GPD2*), and *carnitine palmitoyltransferase 1 A* (*CPT1A*) mRNA expression levels were unaltered by diet in 12-month-old mice (Fig. 3C). Conversely, MR significantly increased hepatic mRNA expression of *PPAR*γ, *CD36,* and *adipose triglyceride lipase* (*ATGL*) in 12-month-old mice (Fig. 3C), but expression levels of the gluconeogenic genes; *G6P* and *PEPCK* were unaltered by diet (Fig. 3C). Methionine restriction diet produced similar changes in hepatic gene expression in 2-month-old mice (Fig. 3D), however, had no effect on *ACC2* or *PPAR*γ (Fig. 3D). These data indicate that MR induces gene expression alterations that reduce hepatic lipid storage and promote fatty acid oxidation.

### MR improves peripheral insulin signaling

Available evidence supports the view that MR increases metabolic flexibility and overall *in vivo* insulin sensitivity (Hasek *et al*., 2010, 2013); however, the effects of dietary MR on tissue-specific insulin sensitivity have not been examined.

**Figure 4:**
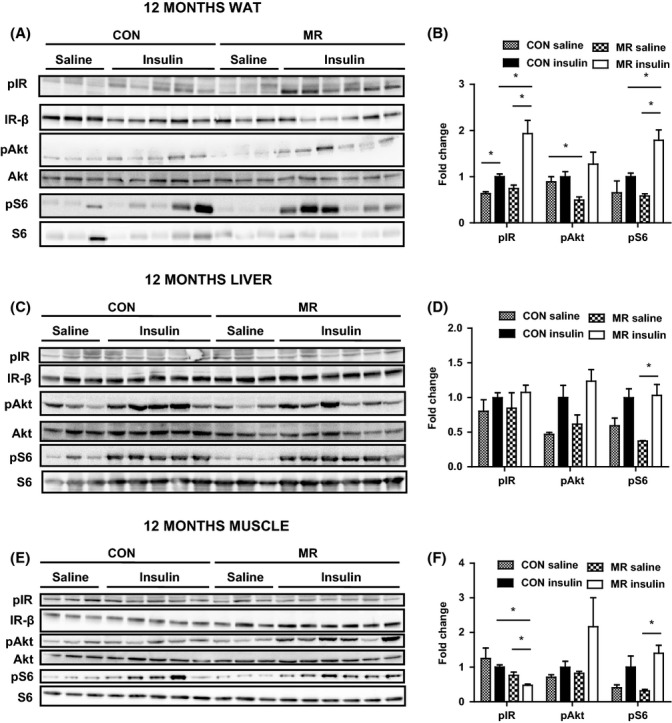
Peripheral insulin signaling in 12-month-old mice on methionine restriction (MR) and control diet. Insulin signaling was assessed by administering either a saline (*n* = 3) or low dose of insulin (0.8 mU g^−1^) (*n* = 5–6) to mice, via i.p. injection, after a 5-h fast. Levels of phosphorylated IR (tyr 1162/1163), protein kinase B/Akt (ser473), S6 (ser235/236), IR-β, total Akt, and total S6 were measured by immunoblotting in (A) epididymal white adipose tissue (WAT), (C) liver, and (E) gastrocnemius muscle in 12-month-old mice fed MR or control diet. Immunoblots were normalized to Ponceau S and total protein in (B) epididymal WAT, (D) liver, and (F) gastrocnemius muscle in 12-month-old mice fed MR or control diet. Data were analyzed as fold change relative to control-fed insulin-injected mice. Significance was calculated by two-tailed Student’s *t*-test (*P < 0.05). Data are represented as mean ± SEM. Black hatch bars, 12-month-old control-fed mice injected with saline; black bars, 12-month-old control-fed mice injected with insulin; white crossed bars, 12-month-old MR-fed mice injected with saline; white bars, 12-month-old MR-fed mice injected with insulin.

To assess insulin-dependent signaling, mice were injected with saline or a physiological dose of insulin (0.8 mU g^−1^) after a 5-h fast. The first tissue examined was epididymal WAT. In 12-month-old mice, both control and MR diet significantly increased levels of phosphorylation of the IR from basal levels upon a bolus of insulin (Fig. 4A,B). In comparison of diets, MR-fed mice had significantly higher levels of insulin-stimulated phosphorylation of the IR than 12-month-old control-fed mice (Fig. 4A,B). Basal phosphorylation of PKB/Akt was significantly reduced by MR relative to control diet in 12-month-old mice (Fig. 4A,B), and MR diet produced an increase from basal to insulin-stimulated levels of phosphorylation of PKB/Akt (*P* = 0.08) (Fig. 4A,B). Insulin-stimulated phosphorylation of S6 was significantly higher in MR diet compared with control diet in 12-month-old mice, and MR significantly enhanced basal to insulin-stimulated levels of phosphorylation of S6 (Fig. 4A,B).

In 2-month-old mice, MR significantly increased levels of IR phosphorylation from basal to insulin stimulation and significantly enhanced levels of insulin-stimulated IR phosphorylation relative to control diet (Fig. S1A,B, Supporting information).

Next, hepatic insulin signaling was examined in the same way as epididymal WAT in 12-month-old mice (Fig. 4C,D). There was no effect of diet on phosphorylation levels of IR or PKB/Akt (Fig. 4C,D). Methionine restriction diet in 12-month-old mice led to a significant increase in phosphorylation of S6 from basal to insulin-stimulated conditions (Fig. 4C,D). In 2-month-old mice, MR produced a significant increase in levels of phosphorylation of Akt and S6 between basal and insulin-stimulated conditions (Fig. S1C,D). In 2-month-old control-fed mice, this only occurred with levels of phosphorylation of S6 (Fig. S1C,D).

Furthermore, insulin signaling was investigated in the gastrocnemius muscle in 12-month-old mice (Fig. 4E,F). Levels of insulin-stimulated phosphorylation of the IR were decreased by MR diet in comparison with the control diet if corrected to total levels (Fig. 4E,F). However, it is important to stress that there is an increase in total level of IR itself, probably reflecting the fact that these mice are more insulin sensitive due to decreased adiposity (Fig. 4E). There was no effect of diet on phosphorylation levels of PKB/Akt (Fig. 4E,F). In 12-month-old mice, MR significantly increased levels of phosphorylation of S6 from basal to insulin stimulation (Fig. 4E,F). In 2-month-old mice, MR increased insulin-stimulated levels of phosphorylation of Akt compared with control diet, and MR also significantly enhanced basal to insulin-stimulated levels of phosphorylation of Akt and S6 (Fig. S1E,F).

### MR increases serum and hepatic gene expression of FGF21 and associated targets

**Figure 5:**
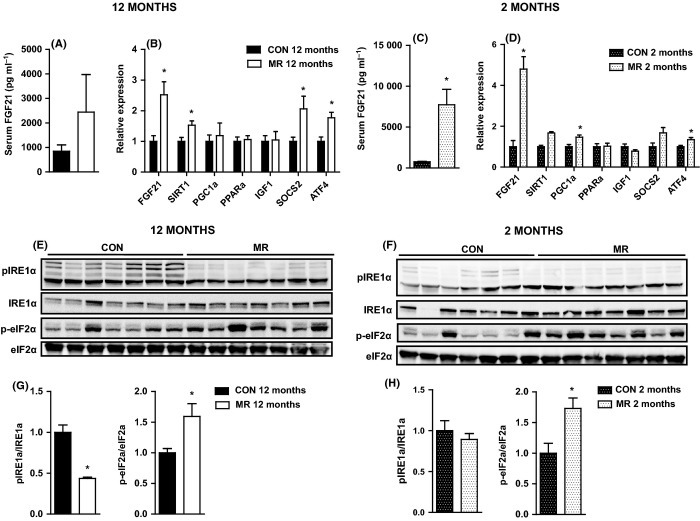
Liver FGF21, ATF4, IRE1α, and eIF2α in mice on methionine restriction (MR) and control diet. (A) Serum FGF21 in 12-month-old mice (*n* = 7), (B) hepatic gene expression of *FGF21* and genes related to FGF21 in 12-month-old mice (*n* = 5–8), (C) serum FGF21 in 2-month-old mice (*n* = 7), and (D) hepatic gene expression of *FGF21* and genes related to FGF21 in 2-month-old mice (*n* = 4–6) fed MR or control diet. Levels of phosphorylated IRE1α, eIF2α, total IRE1α, and eIF2α were measured by immunoblotting in liver after low dose of insulin stimulation (0.8 mU g^−1^) in (E) 12-month-old mice (*n* = 7) and (F) 2-month-old mice (*n* = 7) fed MR or control diet. Immunoblots were normalized to total protein in (G) 12-month-old mice and (H) 2-month-old mice fed MR or control diet. Data for gene expression and immunoblots were analyzed as fold change relative to control-fed mice. Significance was calculated by two-tailed Student’s *t*-test (*P < 0.05). Data are represented as mean ± SEM. Black bars, 12-month-old control-fed mice; white bars, 12-month-old MR-fed mice; black hatch bars, 2-month-old control-fed mice; white hatch bars, 2-month-old MR-fed mice.

FGF21 improves hepatic steatosis and insulin sensitivity in obese mice by stimulating lipolysis and fatty acid oxidation and reducing gluconeogenesis (Xu *et al*., 2009a). Methionine restriction increased levels of serum FGF21 by 2.5-fold in 12-month-old mice (Fig. 5A) and significantly increased hepatic gene expression of *FGF21* (Fig. 5B).

FGF21 induction in the liver is linked to the SIRT1-PGC-1α-PPARa pathway (Purushotham *et al*., 2009), and we found *SIRT1* to be significantly increased by MR in 12-month-old mice; however, *PGC1*α and *PPAR*α were unchanged (Fig. 5B). FGF21 suppresses the GH/IGF-1 signaling axis in liver (Zhang *et al*., 2012); however, MR had no effect on *IGF-1* expression in 12-month-old mice, but, *SOCS2*, an inhibitor of the GH-signaling cascade, was significantly increased by MR (Fig. 5B).

In 2-month-old mice, MR significantly increased serum levels of FGF21 and hepatic expression of *FGF21* and *PGC1*α*,* but had no effect on *SIRT1*, *PPAR*α, *IGF-1,* or *SOCS2* mRNA expression (Fig. 5C,D).

### MR increases hepatic eIF2α phosphorylation and ATF4 gene expression

Amino acid limitation has been shown to activate the PERK arm of ER stress signaling via eukaryotic translation initiation factor 2α (eIF2α) phosphorylation and induction of activating transcription factor 4 (ATF4) (Harding *et al*., 2003); therefore, we went on to examine these markers in the liver.

Methionine restriction significantly upregulated mRNA expression of *ATF4* (Fig. 5B) and significantly increased levels of phosphorylation of eIF2α (Fig. 5E,G) in 12-month-old mice. To ensure it was not an induction of the entire UPR, we examined the first component of another of the UPR branches; IRE1α. Methionine restriction significantly decreased insulin-stimulated IRE1α phosphorylation in 12-month-old mice (Fig. 5E,G).

In 2-month-old mice MR significantly increased *ATF4* mRNA expression (Fig. 5D) and levels of eIF2α phosphorylation (Fig. 5F,H); however, dietary treatment had no effect on levels of phosphorylation of IRE1α (Fig. 5F,H).

### MR increases FGF21 levels and improves glucose tolerance after 48 h of treatment

**Figure 6:**
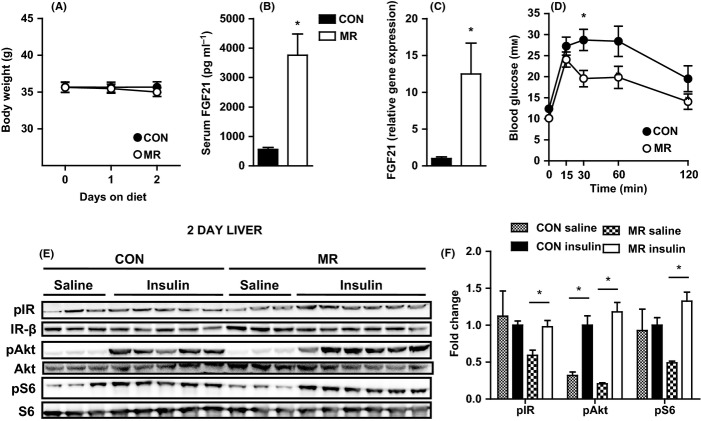
Effects of 48-h methionine restriction (MR) treatment on body weight, FGF21 and glucose homeostasis. (A) Body weight in mice fed MR or control diet (*n* = 13). Significance was calculated by repeated measures two-way ANOVA with Bonferroni multiple comparison *post hoc* tests (*P < 0.05). (B) Serum FGF21 (*n* = 13) and (C) hepatic gene expression of *FGF21* (*n* = 6) in mice fed MR or control diet. Data for gene expression were analyzed as fold change relative to control-fed mice. Significance was calculated by two-tailed Student’s *t*-test (*P < 0.05). (D) Glucose tolerance as assessed by a glucose tolerance test (dose of glucose = 2 g kg^−1^) after a 5-h fast in mice fed MR or control diet (*n* = 9–10). Significance was calculated by repeated measures two-way ANOVA with Bonferroni multiple comparison *post hoc* tests (*P < 0.05). Black bars/circles, control-fed mice; white bars/circles, MR-fed mice. Insulin signaling was assessed by administering either a saline (*n* = 3) or high dose of insulin (10 mU g^−1^) (*n* = 5–6) to mice, via i.p. injection, after a 5 h fast. (E) Levels of phosphorylated IR (tyr 1162/1163), protein kinase B/Akt (ser473), S6 (ser235/236), IR-β, total Akt, and total S6 were measured by immunoblotting in liver in mice fed MR or control diet. (F) Immunoblots were normalized to Ponceau S and total protein in liver in mice fed MR or control diet. Data were analyzed as fold change relative to control-fed insulin-injected mice. Significance was calculated by two-tailed Student’s *t*-test (*P < 0.05). Data are represented as mean ± SEM. Black hatch bars, control-fed mice injected with saline; black bars, control-fed mice injected with insulin; white crossed bars, MR-fed mice injected with saline; white bars, MR-fed mice injected with insulin.

To examine metabolic effects of MR before the effects on body weight, mice were fed MR or control diet for 48 h. After 48 h, there was no change in body weight (Fig. 6A), but serum levels of FGF21 were significantly increased by over sevenfold (Fig. 6B) and hepatic gene expression of FGF21 was significantly increased by over 12-fold by dietary MR (Fig. 6C). Methionine restriction improved glucose tolerance after just 48 h, as assessed in a glucose tolerance test (GTT), with a significant decrease in blood glucose levels at 30 min postinjection (Fig. 6D). Furthermore, after 48 h, MR induced a significant increase in phosphorylation of the IR and S6 from basal to insulin-stimulated levels in the liver (Fig. 6E,F), whereas both MR and control diet increased levels of phosphorylation of PKB/Akt from basal to insulin stimulation (Fig. 6E,F). Therefore, MR is able to induce hepatic FGF21 expression and insulin sensitivity and improve whole-body glucose homeostasis, prior to any changes in body weight.

## Discussion

Aging causes fat accumulation (Huffman & Barzilai, 2009) and dampens insulin sensitivity and whole-body glucose homeostasis (Selman & Withers, 2011), as apparent in this study in the differences we observed between 2- and 12-month-old control-fed mice. We found aging to lead to increased body weight and adiposity and diminished glucose tolerance and insulin sensitivity, associated with increased fasting serum insulin and leptin levels.

Dietary manipulation in the form of MR has been shown before to dramatically decrease body weight and adiposity and improve insulin sensitivity in young, healthy mice (Hasek *et al*., 2010, 2013; Perrone *et al*., 2010; Plaisance *et al*., 2010). We have shown here for the first time that MR is able to reverse aging-associated alterations in physiology, specifically to decrease body fat, increase physical activity, and improve glucose and lipid homeostasis to the levels measured in young mice (fed a normal diet). The increase in age-related adiposity is linked to enhanced risk of diseases and reduced lifespan (Huffman & Barzilai, 2009). Therefore, the ability of MR to reverse age-induced insulin resistance and dysfunctional lipid metabolism, back to levels of healthy young mice, may play a role in its ability to extend lifespan.

This study strengthens the translational evidence for MR as an effective intervention feasible in humans, and treatments that are not only capable of preventing but also treating metabolic dysfunction in adults are urgently needed, due to the rising prevalence of metabolic syndrome (Ford *et al*., 2004). The fact that MR allows greater food consumption, while simultaneously decreasing body weight and adiposity, makes it a more sustainable option than dietary restriction. Methionine restriction produced similar beneficial metabolic effects when begun at 12 months compared with 2 months of age, suggesting that initiating MR at an early age may not be necessary for positive effects on longevity.

In 12-month-old mice, MR was able to produce an increase in lipogenic and oxidative gene expression in epididymal WAT and decrease in lipogenic but increase in lipolytic gene expression in the liver, consistent with lipid homeostasis remodeling shown previously by MR in young mice (Perrone *et al*., 2010; Hasek *et al*., 2013). These changes are likely to account for the decrease in serum and hepatic triglycerides produced by MR, consistent with earlier studies (Ables *et al*., 2012; Hasek *et al*., 2013).

Improvements in whole-body glucose homeostasis with MR have been shown before, but tissue-specific insulin sensitivity at the protein level has not been examined. The major effects of MR on insulin signaling occurred in epididymal WAT; however, hepatic and muscle insulin signaling also showed moderate improvements. Within components of the insulin signaling pathway, the significant increase from basal to insulin-stimulated levels of phosphorylation, produced by MR, expresses an enhanced ability of peripheral tissues to respond to a bolus of insulin. These findings support those from previous studies, which found MR to increase metabolic flexibility, shown through an enhanced range of respiratory quotient from day to night (Hasek *et al*., 2010).

Previous results have also found MR to increase circulating (Ables *et al*., 2012) and hepatic gene expression of FGF21 (Ables *et al*., 2012; Perrone *et al*., 2012) and one of its target genes, *PGC1*α (Perrone *et al*., 2010). Although FGF21 could be induced by a PPARα mechanism (Badman *et al*., 2007), due to upregulation of SIRT1 and PGC1α, PPARα was unchanged. An alternative is that FGF21 is induced in the liver under leucine deprivation through the general control nonderepressible 2 (GCN2) – phospho-eIF2α – ATF4 pathway (De Sousa-Coelho *et al*., 2012). Dietary MR resulted in enhanced levels of phosphorylation of eIF2α and substantial upregulation of hepatic *ATF4* mRNA expression, suggesting activation of GCN2. In addition, the reduction in levels of phosphorylation of IRE1α in 12-month-old mice ensures that the induction of eIF2α was not representative of activation of the entire UPR and that it was only this specific branch that responds to amino acid deficiency (De Sousa-Coelho *et al*., 2012). Moreover, it provides evidence that MR can reduce specific aspects of ER stress while increasing the plausibility that FGF21 is induced in the same way in response to MR as it is upon leucine deprivation.

FGF21 upregulation after just 48 h of MR feeding preceded any change in body weight, suggesting that FGF21 could be responsible for the effects of MR on body weight and adiposity. Indeed, FGF21 has dramatic effects to reverse obesity in high-fat diet-induced obese mice (Xu *et al*., 2009a). The improvements in whole-body glucose homeostasis could conceivably be secondary to MR decreasing adiposity and body weight; however, after 48 h of MR feeding, glucose tolerance and hepatic insulin sensitivity were also improved. This provides supporting evidence for MR’s effects on insulin sensitivity not being entirely secondary to body weight loss and there being another contributing factor. The improvement was in line with FGF21 induction, and FGF21 has been shown to have insulin-sensitizing effects independently of body weight alterations, including significantly enhanced hepatic and peripheral insulin sensitivity and restored glucose tolerance in diet-induced obese mice (Xu *et al*., 2009a).

FGF21 could play a role in the metabolic effects of MR in the liver, where decreased lipogenic gene expression and lipid storage was present in MR-fed animals. FGF21 induction in the liver in response to leucine deprivation caused downregulation of hepatic lipogenesis and reduced hepatic lipid accumulation, the effects of which were absent when FGF21 KO mice were deprived of leucine (De Sousa-Coelho *et al*., 2012). Moreover, FGF21 reduces GH/IGF-1 signaling (Zhang *et al*., 2012); therefore, the upregulation of hepatic *SOCS2* expression could also be produced by FGF21.

Adipose tissue is sensitive to circulating FGF21 as it contains high levels of the essential coreceptor, β-Klotho (Fisher *et al*., 2011), which was upregulated by MR. FGF21 in adipose tissue plays a role in thermogenesis and browning of WAT, through activation of UCP1 and PGC1α (Fisher *et al*., 2012), and could explain the upregulation of oxidative gene expression, *PGC1*α and *TFAM*, in response to MR in epididymal WAT and earlier findings of MR increasing energy expenditure and WAT UCP1 levels (Hasek *et al*., 2010).

Interestingly, FGF21 also produces a lipid futile cycle in WAT (Coskun *et al*., 2008), which agrees with the results of this study and others, where MR produced a simultaneous increase in lipogenic and oxidative gene expression in epididymal WAT (Perrone *et al*., 2008, 2010, 2012; Hasek *et al*., 2013). FGF21 also increases glucose uptake into WAT (Xu *et al*., 2009b), which could explain the effects of MR to significantly increase insulin-stimulated phosphorylation of the IR and S6 compared with control diet. In addition, FGF21 has been shown to cause hyperphagia (Coskun *et al*., 2008), which could explain the increased food intake by MR-fed animals.

In conclusion, we present the data that MR is able to reverse age-induced metabolic dysfunction in fully developed adult mice, back to levels of young, healthy mice on a normal diet. This could explain the increased lifespan of mice when MR diet was begun at 12 months of age (Sun *et al*., 2009). In addition, like MR, FGF21 treatment extends lifespan in mice (Zhang *et al*., 2012) and FGF21 could be the basis of the mechanism behind MR’s effects to reverse age-induced dysfunctional metabolism, through the remodeling of hepatic and WAT lipid homeostasis. This study also provides evidence that the effects of MR are not only secondary to its effects on reducing growth and development and that in fully developed and grown mice, it may slow aging.

## Experimental procedures

### Animals

This study was approved by the University of Aberdeen Ethics Review Board and performed under UK Home Office project license PPL 60/3951. Male C57BL/6J wild-type mice (Charles River, Edinburgh, UK) were singly housed. Mice were exposed to 12-h light/dark cycle at 22–24 °C and had *ad libitum* access to food and water. Mice were placed on a control diet containing 0.86% methionine (Dyets, Bethlehem, PA, USA) for 2 weeks. Within each age category, mice were randomized by body weight; half were maintained on control diet and half were switched to MR diet containing 0.172% methionine (Dyets). The glutamic acid content of the MR diet was increased to compensate for the reduced methionine content and to create equal amounts of total amino acids in both diets. Mice were maintained on diets for 8 weeks (or 48 h where indicated) and terminal tissues collected after a 5-h fast plus intraperitoneal (i.p.) injection with saline (154 mm NaCl) or a low, physiological dose of insulin (0.8 mU g^−1^ body weight) and sacrificed after 10 min. Tissues were immediately dissected and frozen in liquid nitrogen after cervical dislocation. In the short-term study, mice were maintained on MR and control diets for 48 h and terminal tissues collected in the same way, with the only difference being in the dosage of insulin (10 mU g^−1^ body weight).

### Whole-body measurements and blood metabolites

Body weight and food intake were measured every 3 days throughout the study. Body composition was measured after 28 days on MR or control diet by dual-energy X-ray absorptiometry (DEXA) (Lunar PIXIMUS-Densitometer; GE Medical Systems, San Francisco, CA, USA). GTTs were performed after 32 days on MR or control diet and involved fasting for 5 h prior to i.p. injection with glucose (2 g kg^−1^ body weight). Tail blood glucose measurements using glucometers (AlphaTRAK, Berkshire, UK) were taken immediately before and 15, 30, 60, and 120 min after i.p. injection with glucose. Tail vein blood samples were taken after a 5 h fast, after 39 days on MR or control diet. All serum measurements were taken on these. Serum leptin (Crystal Chem, Downers Grove, IL, USA), insulin (Crystal Chem), and FGF21 (Millipore, Darmstadt, Germany) were determined by ELISA. Serum glucose was measured by glucose oxidase assay (Thermo Scientific, Waltham, MA, USA). Serum triacylglycerol (17628; Sentinel Diagnostics, Milan, Italy) and serum FFAs (Wako Chemicals, Richmond, VA, USA) were determined using appropriate kits. In the short-term study, tail vein blood samples were taken after mice were sacrificed and these were used to measure FGF21 by ELISA. Another cohort of male C57BL/6J wild-type mice was used to measure glucose tolerance, in the same way as described above, after 48 h of dietary treatment.

### Locomotor activity measurement

Daily locomotor activity was monitored after 45 days on MR or control diet, using passive infrared sensors (Panasonic – AMN11112; Farnell, Leeds, UK) mounted on top of each cage. Clocklab software (Actimetrics, Wilmette, IL, USA) was used to acquire and analyze data generated by the PIR sensors. Animals were given 48 h to acclimatize to the presence of the PIR sensor, before data were recorded from each individual animal every minute for seven consecutive days under 12-h light/dark cycles. Data were expressed as beam breaks per minute and consolidated into 1 h bins for analysis.

### Liver triacylglycerols

Liver pieces (100 mg) were cut and weighed. Each sample was homogenized in 1 mL PBS before being centrifuged for 15 s at room temperature. The top layer was resuspended, and the supernatant fraction was assayed for triacylglycerols using a commercial kit (Sentinel Diagnostics).

### Immunoblotting

Frozen liver and epididymal WAT lysates were prepared in RIPA buffer, as described previously (Delibegovic *et al*., 2007). Proteins were separated by 4–12% SDS-PAGE and transferred to nitrocellulose membranes. Immunoblots were performed using antibodies from Cell Signaling Technology (Cell Signaling by NEB, Hitchin, UK) (unless stated otherwise) against phospho-S6 (s235/236) 4858S; total S6 2217S; phospho-Akt/PKB (s473) 9271S; phospho-eIF2α (s51) 9721S; total eIF2α 5324S; total IRE1α 3294S; phospho-IRE1α (s724) PA1-16927 (Thermo Scientific); phospho-IR (tyr1163/1163) 44804G (Invitrogen, Paisley, UK); phospho-IR (tyr1158) 44802G (Invitrogen); IR-β sc-711 (Santa Cruz, Dallas, TX, USA); total Akt/PKB A2210 (Santa Cruz); and extracellular signal-regulated kinase 2 (ERK2) I2908 (Santa Cruz). Proteins were visualized with enhanced chemiluminescence and quantified using Bio-1D software (Peqlab, Sarisbury Green, UK).

### Gene expression in liver and epididymal WAT

Total RNA was isolated from frozen liver and epididymal WAT using peqGOLD TriFast (Peqlab). For epididymal WAT, homogenates were centrifuged and had the lipid layer removed before phase separation. cDNA was synthesized from 1 μg RNA using bioscript cDNA synthesis kit (Bioline, London, UK), oligo (dT) 18 primers and random hexamer primers. Target genes were amplified by quantitative PCR (qPCR), using gene-specific primers and GoTaq qPCR master mix (Promega, Southampton, UK) on the LightCycler-480 (Roche, Burgess Hill, UK). Relative mRNA levels were calculated using the Pfaffl method (Pfaffl, 2001) and normalized to a geometric mean of three reference genes; *beta-actin*, *hypoxanthine-guanine phosphoribosyltransferase* (*HPRT*), and *Ywhaz* for the liver, and *Nono*, *Ywhaz,* and *HPRT* for epididymal WAT.

### Statistical analysis

Data are expressed as mean ± SEM. Statistical analyses were performed using repeated measures two-way ANOVA with Bonferroni multiple comparison *post hoc* tests, one-way ANOVA with Tukey’s *post hoc* test, or two-tailed Student’s t-tests, as appropriate. graphpad prism 5 software (GraphPad Software, Inc., San Diego, CA, USA) was used for analyses. *P*-values < 0.05 were considered significant.

## Supporting Information

**Fig. S1** Peripheral insulin signaling in 2-month-old mice on MR and control diet.
